# Adherence to the Mediterranean diet, inflammatory biomarkers and cognitive status in older Italian adults

**DOI:** 10.3389/fnut.2026.1809163

**Published:** 2026-04-29

**Authors:** Margherita Grasso, Francesca L’Episcopo, Annamaria Fidilio, Marco Antonio Olvera-Moreira, Giuseppe Toscano, Stefano Muratore, Margherita Drago, Sabrina Musso, Veronica Bentivegna, Lucrezia Costanzo, Melannie Toral-Noristz, Raynier Zambrano-Villacres, Lisandra León Brizuela, Giuseppe Lanza, Raffaele Ferri, Filippo Caraci

**Affiliations:** 1 Oasi Research Institute-IRCCS, Troina, Italy; 2 Department of Drug and Health Sciences, University of Catania, Catania, Italy; 3 Carrera de Medicina, Universidad Católica de Santiago de Guayaquil, Guayaquil, Ecuador; 4 Primary Health Care Unit, Provincial Health Authority of Enna, Enna, Italy; 5 Escuela de Medicina, Universidad Espíritu Santo, Samborondón, Ecuador; 6 Facultad de Ciencias de la Salud y Desarrollo Humano, Universidad Ecotec, Samborondón, Ecuador; 7 Universidad Internacional Iberoamericana, Campeche, Mexico; 8 Universidade Internacional do Cuanza, Cuito, Bié, Angola; 9 Fundación Universitaria Internacional de Colombia, Bogotá, Colombia; 10 Department of Surgery and Medical-Surgical Specialties, University of Catania, Catania, Italy

**Keywords:** diet quality, inflammation, Mediterranean diet, mild cognitive impairment, neuroinflammation

## Abstract

**Background:**

Mild Cognitive Impairment (MCI) is viewed as a transitional stage between normal brain aging and dementia and is characterized by subtle cognitive deficits without significant impairment in daily functioning. Growing evidence supports the contribution of neuroinflammation and modifiable lifestyle factors, including diet, in the progression of cognitive decline.

**Aim:**

This study aimed to investigate the association between adherence to the Mediterranean diet, neuroinflammatory biomarkers, and MCI status in older adults.

**Design:**

Ninety-two participants were enrolled in this cross-sectional study, including 37 subjects with MCI. Dietary intake was assessed using a validated food frequency questionnaire (FFQ) and adherence to the Mediterranean diet explored through the MedDietScore. Plasma levels of TGF-β1 and TNF-α were measured by ELISA. Cognitive status was evaluated using the Mini Mental Examination (MMSE) and the Montreal Cognitive Assessment (MoCA), both adjusted for age and education. Statistical analyses included non-parametric tests, correlation analysis, and logistic regression models.

**Results:**

MCI patients showed significantly reduced plasma levels of TGF-β1 and increased TNF-α concentrations compared to other participants. After adjustment for potential confounding factors, greater adherence to the Mediterranean diet was associated with a lower likelihood of MCI in a dose–response manner (highest versus lowest adherence quartile, odds ratio: 0.07, 95% confidence interval: 0.01–0.60). Additional adjustment for inflammatory biomarkers attenuated the associations, suggesting a potential mediating role.

**Conclusion:**

Our findings showed that higher adherence to Mediterranean diet is associated with lower likelihood of being MCI. Such a relation might be, at least in part, mediated by inflammatory biomarkers. Overall, these results support the role of dietary modulation in preventive strategies against cognitive decline and progression into MCI.

## 1 Introduction

Globally, demographic aging is proceeding at an unprecedented pace (1). The United Nations estimates that by 2050, the number of individuals aged 65 years and older will more than double from approximately 727 million in 2020 to around 1.5 billion, substantially increasing the proportion of older adults within the total population (2). This demographic shift has direct implications for age-associated health conditions, particularly cognitive disorders, which constitute a major public health and socioeconomic burden (1).

Dementia, an umbrella term for progressive neurocognitive disorders characterized by significant impairment in memory, executive function, language, or behavior, affects about 55 million people worldwide (3). Projections indicate this number will escalate to 78 million by 2030 and 139 million by 2050, largely due to population aging and associated risk factors (3). Over 60% of individuals with dementia currently reside in low- and middle-income countries, a proportion expected to rise to approximately 71% by 2050 (4). These trends underscore the increasing global impact of cognitive decline on healthcare systems and societies (5).

Mild cognitive impairment (MCI) represents a distinct clinical entity defined by objective cognitive decline greater than expected for an individual’s age and educational attainment, yet insufficient to meet criteria for dementia and generally preserving functional independence (6). MCI is considered a transitional stage between normal cognitive aging and dementia, with variable and heterogenous prognoses (6). Epidemiological studies report that the prevalence of MCI among community-dwelling adults aged 50 years and older lies in the range of approximately 15% worldwide, with variation by diagnostic criteria, age strata, and population characteristics (7). Estimates specifically in geriatric populations (typically defined as ≥ 60 years) suggest a prevalence of around 24%, highlighting the substantial frequency of MCI in older cohorts (8).

Given the high and increasing prevalence of MCI and dementia in aging populations, identifying modifiable factors that may preserve cognitive function or delay progression along the cognitive continuum has become a research priority (9). Among lifestyle factors, dietary patterns have garnered significant interest due to their influence on systemic inflammation, activation of the endogenous antioxidant defense system and a modulation of the gut microbiota (10). The Mediterranean diet is a dietary pattern characterized by high intake of fruits, vegetables, legumes, whole grains, nuts, and olive oil; moderate consumption of fish and wine; and low intake of red and processed meats and saturated fats (11). Such a pattern has been associated with reduced risk of cardiovascular disease, metabolic disorders, mortality (12) and overall quality of life (13). These beneficial effects have been hypothesized to be at least partially mediated through the reduction of systemic inflammation (14), a known etiology of various chronic diseases, including cognitive disorders (15). Increasing evidence supports a key role of neuroinflammation in dementia progression (16), with sustained and enhanced inflammatory responses that increase the conversion from MCI into Alzheimer ’s disease (AD)-type dementia (17). Hence, the potential neuroprotective effects of diet have been increasingly investigated in the context of cognitive aging. In fact, increasing scientific evidence hypothesized that dietary factors may target pathological processes involved in cognitive decline development, including neuroinflammation, impaired neurogenesis, and synaptic plasticity, thus preventing or controlling its manifestation. Adequate nutrition could in fact modulate the immune system response and the neuroinflammatory processes involved in the pathogenesis of AD-related cognitive decline and in the neurodegenerative progression (18, 19).

Emerging evidence from observational studies suggests that higher adherence to the Mediterranean diet is associated with lower risk of cognitive impairment, dementia and Alzheimer’s disease (20). Evidence has also shown that adherence to a healthy diet, such as the Mediterranean diet, could be an important modifiable risk factor for healthy brain aging, supporting the hypothesis of a relationship between the Mediterranean diet and age-related cognitive functions (19). Interestingly, while observational studies suggest that higher adherence to the Mediterranean diet is associated with a lower likelihood of being MCI among cognitively normal individuals (21), recent clinical trials showed rather disappointing results of the effects of dietary interventions promoting a Mediterranean-style diet in MCI patients and cognitive function (22, 23). The potential limitation of such an experimental approach is represented by a relatively short duration of a clinical intervention as compared to a culturally embedded lifestyle naturally adopted by certain individuals living in a Mediterranean island, most likely for their entire lives. Proposed biological mechanisms include reduced oxidative stress phenomena, attenuation of chronic inflammation, favorable modulation of lipid profiles and glucose metabolism, and preservation of cerebrovascular integrity (24, 25). However, heterogeneity in study designs and baseline characterization of both cognitive status and dietary habits necessitates further investigation, particularly regarding early cognitive states such as MCI. The present study aims to examine the association between adherence to the Mediterranean diet and cognitive status, with a primary focus on the likelihood of MCI. Clarifying this relationship may inform dietary recommendations and public health strategies targeting cognitive health in aging populations.

## 2 Materials and methods

### 2.1 Study population

Consecutive patients undergoing a standard evaluation at the Geriatrics Unit of the IRCCS Oasi Research Institute-IRCCS in Troina (Italy) were considered as potential participants in the study. Eligible participants were adults older than 65 years presenting with subjective memory impairment. Exclusion criteria comprised any other dementia subtype, dementia related to neurological conditions, and the presence of psychiatric disorders. A total of 92 patients were finally recruited in the study. The present study was conducted according to the Declaration of Helsinki, the Guidelines for Good Clinical Practice and it was approved by the Ethics Committee of the Oasi Research Institute-IRCCS in Troina (Italy) (CEL-IRCCS OASI/10-04-2025/04; Verbale n. 9 del 19/10/2023, Codice Locale Progetto: T5-AN-11).

### 2.2 Cognitive function assessment

Global cognitive performance was assessed using two standardized screening instruments: the Mini Mental State Examination (MMSE) (26, 27) and the Montreal Cognitive Assessment (MoCA) (28). The MMSE is a 30-item clinician-administered questionnaire designed to provide a brief quantitative measure of overall cognitive status. It evaluates multiple cognitive domains including temporal and spatial orientation (10 points), immediate and delayed memory (6 points), attention and calculation (5 points), language abilities (8 points), and visuoconstructive skills (1 point). Items consist of a combination of verbal questions and simple tasks, with dichotomous or short open responses scored as correct or incorrect. Total scores range from 0 to 30, with higher scores indicating better cognitive functioning. Conventional interpretative thresholds were applied, with scores below normative cut-offs (adjusted for age and educational level) considered indicative of possible cognitive impairment. The MoCA is also a 30-point screening tool specifically developed to detect mild forms of cognitive decline. It consists of 12 items grouped into several domains: visuospatial/executive function (5 points), naming (3 points), attention and working memory (6 points), language (3 points), abstraction (2 points), delayed recall (5 points), and orientation (6 points). Responses include multiple-choice, drawing tasks, and short verbal answers. As recommended by the original guidelines, one additional point was added to the total score for individuals with ≤ 12 years of formal education. Scores range from 0 to 30, with higher scores reflecting better performance; values below established normative thresholds were interpreted as suggestive of cognitive impairment. The MoCA was included to increase sensitivity for subtle cognitive deficits, particularly in executive and attentional domains that are often affected in early stages of impairment.

### 2.3 Diagnosis of mild cognitive impairment

Classification of participants as cognitively normal or affected by mild cognitive impairment (MCI) was based on criteria consistent with the Diagnostic and Statistical Manual of Mental Disorders, Fifth Edition (DSM-5). Diagnosis required the presence of: (i) evidence of a modest decline in one or more cognitive domains relative to previous functioning, documented through performance on standardized tests (MMSE and/or MoCA scores below age- and education-adjusted norms); (ii) preservation of functional independence in basic activities of daily living, as determined through clinical interview; (iii) absence of significant interference with social or occupational functioning; and (iv) exclusion of major neurocognitive disorder, neurological disease, or psychiatric conditions that could better account for the cognitive deficits. Final diagnostic attribution was made by experienced clinicians based on the combination of test results and clinical evaluation.

### 2.4 Assessment of clinical and brain-related conditions

To control for potential non-cognitive factors influencing neuropsychological performance, all participants were evaluated using validated clinical screening instruments. Depressive symptoms were assessed through two complementary scales. The Hamilton Depression Rating Scale (HDRS) (29) is a clinician-rated instrument composed of 17 core items assessing mood, guilt, insomnia, anxiety, psychomotor changes, and somatic symptoms. Each item is scored on either a 3- or 5-point Likert-type scale, yielding a total score ranging from 0 to 52, with higher scores indicating greater depressive severity. In addition, the Geriatric Depression Scale—short form (GDS-SF), a 15-item self-report questionnaire specifically developed for older adults, was administered (27, 30). The GDS-SF uses a dichotomous “yes/no” response format, with total scores ranging from 0 to 15; higher scores reflect more pronounced depressive symptomatology. The combined use of HDRS and GDS allowed both clinician-based and self-perceived assessment of mood symptoms in the elderly population. Daytime sleepiness was evaluated using the Epworth Sleepiness Scale (ESS) (31). The ESS is an 8-item self-administered questionnaire that measures the individual’s usual chance of dozing in common daily situations. Each item is rated on a 4-point Likert scale from 0 (“would never doze”) to 3 (“high chance of dozing”). Total scores therefore range from 0 to 24, with higher scores indicating greater levels of daytime somnolence and possible sleep-related disturbances.

### 2.5 Dietary assessment

Dietary information was collected using a food frequency questionnaire (FFQ) previously validated for the Sicilian population (32). Participants were asked to report their usual dietary habits by indicating how often, on average, they had consumed each item from a list of 110 commonly eaten foods and beverages during the previous year. For each food item, nine response options were provided, ranging from “never or almost never” to “4–5 times per day,” allowing a detailed estimation of habitual intake frequency. Standard portion sizes included in the FFQ were used to estimate the amount of food consumed at each eating occasion. Reported frequencies were converted into average daily intakes through established algorithms, enabling the calculation of mean consumption in grams or milliliters per day for each food item. Nutrient and energy intakes were subsequently estimated by linking individual consumption data to the Italian Food Composition Tables of the Research Center for Food and Nutrition (CREA). These tables were used as the reference source for determining the caloric content as well as the macro- and micronutrient composition of all reported foods. For each participant, total daily energy intake and nutrient profiles were calculated by multiplying the nutrient content of each food, as provided in the composition tables, by its corresponding estimated daily consumption. This procedure allowed the derivation of quantitative estimates of overall dietary intake, including total calories, macronutrients, vitamins, minerals, and other dietary components.

Adherence to the Mediterranean diet was evaluated using a Mediterranean Diet Score adapted from the index originally proposed by Panagiotakos et al. (33). This score is based on self-reported consumption of alcohol and 10 food groups representative of the Mediterranean dietary pattern. For food groups considered characteristic of the Mediterranean diet (including fruits, vegetables, legumes, olive oil, fish, potatoes, and non-refined cereals), higher scores were assigned to higher consumption frequencies. A maximum score of 5 was attributed to daily or more frequent consumption, with proportionally lower scores assigned to less frequent intake, and a score of 0 assigned to rare or no consumption. Conversely, for food groups considered inconsistent with the Mediterranean dietary pattern, such as red meat and meat products, the scoring scheme was reversed, with higher scores assigned to lower consumption frequencies. Alcohol intake was quantified by converting reported servings into milliliters of alcohol per day. Moderate alcohol consumption received higher scores, whereas abstention or high intake was assigned lower scores. Specifically, intakes below 300 mL/day were assigned the highest score, with progressively lower scores assigned to increasing levels of consumption, and a score of 0 assigned to intakes exceeding 700 mL/day or to no alcohol consumption. Component scores were summed to obtain an overall Mediterranean diet score ranging from 0 to 55, with higher values indicating greater adherence to the Mediterranean dietary pattern.

### 2.6 Markers of inflammation

Plasma samples obtained from the participants at the time of the initial neuropsychological evaluation (T0) were collected according to standard procedures as previously described (34). Briefly, venous blood samples were collected in lavender EDTA-K2 BD Vacutainer tubes and centrifuged at 1,900 rpm for 10 min to separate the plasma from cellular components. After a second centrifugation (3,900 rpm, 10 min), the plasma samples were aliquoted and stored at −80° until use. Plasma concentrations of TGF-β1 ng/ml and TNF-α pg/ml were measured by enzyme-linked immunosorbent assay (ELISA). The quantitative determination of active human TGF-β1 in diluted plasma samples (1:10), was evaluated according to manufacturer’s instructions (Bio-Techne, R&D system, cod. DB100C). The plasmatic concentrations of TNF-α were determined using the human TNF-α ELISA kit (Bio-Techne, R&D system, cod. DTA00D) following the protocol detailed by the manufacturer. The optical density of each well was determined using a Varioskan LUX Multimode Microplate Reader (Agilent BioTek, Santa Clara, CA, United States) set to 450, 540, and 570 nm. Subtract readings at 540 or 570 nm from the readings at 450 nm were performed to correct the optical imperfections in the plate. We then analyzed the TGF-β1/TNF-α ratio in order to create a composite biomarker in order to characterize a novel model adaptable for assessing the early-stage of cognitive decline. The TGF-β1/TNF-α ratio was calculated considering the plasmatic concentration of TGF-β1 pg/mL and TNF-α pg/mL.

### 2.7 Statistical analysis

Categorical variables are presented as frequency of occurrence and percentage with differences between groups tested through Chi-square test. Continuous variables are presented as mean and standard deviations, with differences between groups tested through Student’s *t*-test and Mann-Whitney U tests as parametric and non-parametric tests of normally and not normally distributed variables, respectively. The association between overall adherence to the Mediterranean diet and the likelihood of mild cognitive impairment was evaluated using logistic regression models to calculate odds ratios (ORs) and corresponding 95% confidence intervals (CIs) for the association of quartiles of Mediterranean Diet Score as the exposure and MCI as the outcome. In order to test for potential confounding or mediating factors, three hierarchical models were fitted. Model 1 was adjusted for total energy intake. Model 2 was additionally adjusted for demographic and lifestyle covariates, including age, sex, educational attainment, and smoking status. Model 3 was further adjusted for circulating biomarkers of inflammation (TGF-β1 and TNF-α levels) to explore whether inflammatory pathways may mediate the association between Mediterranean diet adherence and mild cognitive impairment. To assess whether the observed association was driven by any single dietary component, a series of sensitivity analyses was conducted. The Mediterranean Diet Score was recalculated iteratively by sequentially excluding one component at a time while retaining all remaining components. For each modified score, associations with mild cognitive impairment were re-estimated using the same set of hierarchical models. This approach was used to evaluate the robustness of the primary findings and to determine whether any individual dietary component disproportionately influenced the overall association. Statistical significance was deemed as *P* < 0.05. All analyses were performed with SPSS 29 (SPSS Inc., Chicago, IL, United States) software.

## 3 Results

### 3.1 Characteristics of the study sample

A total of 92 older adults were included in the analyses. Participants were categorized into quartiles (Q1–Q4) of adherence to the Mediterranean diet according to the Mediterranean Diet Score (MDS). Background characteristics across quartiles of adherence are presented in Table 1. Overall, the sample was predominantly female (54.3%), with a mean age ranging from 72.1 to 75.9 years across quartiles. No statistically significant differences were observed among quartiles of Mediterranean diet adherence with respect to age (*p* = 0.206), sex distribution (*p* = 0.302), body mass index (*p* = 0.516), educational level (*p* = 0.352), or smoking status (*p* = 0.600), indicating a generally comparable sociodemographic profile across adherence groups.

**TABLE 1:** Background characteristics of the study sample by level of adherence to the Mediterranean diet.

	Mediterranean diet				
	Q1 (*n* = 21)	Q2 (*n* = 19)	Q3 (*n* = 28)	Q4 (*n* = 24)	*P*-value
Sex, n (%)					0.302
Male	7 (33.3)	7 (36.8)	14 (50.0)	14 (58.3)	
Female	14 (66.7)	12 (63.2)	14 (50)	10 (41.7)	
Age, mean (SD)	75.9 (6.8)	73.2 (7.9)	72.07 (6.9)	72.2 (5.3)	0.206
BMI, mean (SD)	29.2 (5.5)	27.2 (3.8)	29.08 (3.8)	27.9 (4.1)	0.516
Educational level, n (%)					0.352
Low	12 (57.1)	7 (36.8)	12 (42.9)	7 (29.2)	
Medium	7 (33.3)	11 (57.9)	15 (53.6)	13 (54.2)	
High	2 (9.5)	1 (5.3)	1 (3.6)	4 (16.7)	
Smoking status, n (%)					0.600
Smoker	9 (47.4)	5 (26.3)	10 (35.7)	9 (39.1)	
Non-Smoker	10 (52.6)	14 (73.7)	18 (64.3)	14 (60.9)	

### 3.2 Cognitive and clinical characteristics by Mediterranean diet adherence

Clinical and cognitive characteristics of the participants according to quartiles of Mediterranean diet adherence are summarized in Table 2. Mean scores on the MMSE ranged from 25.8 ± 3.1 in the lowest adherence quartile to 27.2 ± 2.7 in the highest quartile, without reaching statistical significance (*p* = 0.458). A similar pattern was observed for MoCA scores, which showed a non-significant trend toward higher performance with greater diet adherence (*p* = 0.105). No significant differences across adherence quartiles were found for depressive symptoms assessed by the GDS (*p* = 0.872), HDRS (*p* = 0.590), or daytime sleepiness assessed by the ESS (*p* = 0.726).

**TABLE 2:** Clinical characteristics of the study sample by level of adherence to the Mediterranean diet.

	Mediterranean diet				
	Q1 (*n* = 21)	Q2 (*n* = 18)	Q3 (*n* = 27)	Q4 (*n* = 23)	*P*-value
MMSE, mean (SD)	25.8 (3.05)	26.8 (2.05)	26.8 (3.2)	27.2 (2.7)	0.458
MoCa, mean (SD)	21.2 (3.9)	24.2 (4.1)	23.3 (4.4)	23.9 (4.3)	0.105
GDS, mean (SD)	2.6 (2.5)	3.5 (2.6)	3.08 (3.3)	3.1 (3.3)	0.872
HDRS, mean (SD)	6.1 (2.4)	5.6 (4.8)	4.9 (3.6)	4.7 (3.6)	0.590
ESS, mean (SD)	3.1 (2.1)	3.6 (3.04)	3.1 (3.2)	2.6 (2.4)	0.726

MMSE, Mini-Mental State Examination; MoCa, Montreal Cognitive Assessment, GDS, Geriatric Depression Scale; HDRS, Hamilton Depression Rating Scale; ESS, Epworth Sleepiness Scale.

### 3.3 Dietary intake according to MCI status

Mean intakes of major food groups and nutrients stratified by cognitive status are reported in Table 3. Participants with MCI (*n* = 37) tended to consume lower amounts of several Mediterranean diet–related food groups compared with non-MCI participants (*n* = 55), although most differences did not reach statistical significance. Notably, mean fruit intake was lower in individuals with MCI (413.4 ± 425.3 g/day) than in cognitively unimpaired participants (570.8 ± 459.1 g/day), approaching significance (*p* = 0.100). The only dietary variable that differed significantly between groups was vitamin C intake, which was markedly lower among MCI participants (124.8 ± 111.2 mg/day) compared with non-MCI participants (213.7 ± 219.6 mg/day; *p* = 0.025). A borderline difference was observed for nut consumption (*p* = 0.059), with lower intake among individuals with MCI. No significant group differences were found for olive oil, fish, vegetables, whole grains, total energy intake, or macronutrient composition.

**TABLE 3:** Mean intake of major food groups and nutrients by MCI status in the study sample.

	MCI status		
	No (*n* = 55)	Yes (*n* = 37)	
	Mean (SD)	Mean (SD)	*P*-value
Fruit (g/d)	570.8 (459.1)	413.4 (425.3)	0.100
Vegetables (g/d)	304.2 (315.6)	227.6 (248.3)	0.219
Olive oil (g/d)	9.1 (2.3)	9.4 (1.8)	0.643
Legumes (g/d)	31.8 (33.8)	29.7 (32.09)	0.767
Red meat (g/d)	33.8 (25.6)	26.8 (15.1)	0.140
Processed meat (g/d)	8.9 (11.7)	6.8 (7.1)	0.330
Fish (g/d)	40.9 (30.5)	44.2 (46.8)	0.678
Refined grains (g/d)	95.1 (35.2)	100.6 (40.2)	0.495
Whole grains (g/d)	60.7 (90.5)	32.6 (79.04)	0.128
Total grains (g/d)	155.9 (106.0)	133.2 (112.5)	0.329
Potatoes (g/d)	22.9 (37.3)	22.8 (28.7)	0.996
Dairy (g/d)	204.7 (138.9)	203.3 (183.8)	0.967
Sweets (g/d)	66.2 (97.2)	50.9 (51.9)	0.387
Nuts (g/d)	20.3 (29.3)	10.7 (9.3)	0.059
Alcohol (g/d)	8.4 (11.4)	7.06 (12.6)	0.591
Energy (kcal)	2138.07 (922.5)	1924.1 (743.2)	0.243
Energy (kj)	8760.3 (3851.5)	7859.6 (3113.5)	0.239
Proteins (g/d)	85.2 (32.8)	77.2 (35.1)	0.269
Lipids (g/d)	62.9 (32.1)	53.2 (21.8)	0.111
Cholesterol (mg/d)	160.8 (61.8)	149.2 (65.7)	0.395
Carbohydrates (g/d)	315.1 (146.1)	292.5 (125.7)	0.444
Total fibers (g/d)	36.9 (22.8)	31.2 (26.4)	0.270
Total SFA (%)	23.7 (10.8)	20.9 (9.0)	0.208
Total MUFA (%)	27.4 (12.6)	24.02 (8.4)	0.151
Total PUFA (%)	12.7 (10.6)	9.8 (5.2)	0.134
C18:3 (g/d)	1.6 (1.7)	1.04 (0.5)	0.057
C22:6 (g/d)	0.2 (0.2)	0.2 (0.3)	0.837
Thiamine (mg/d)	1.8 (1.3)	1.4 (0.9)	0.128
Riboflavin (mg/d)	2.3 (1.6)	1.9 (0.9)	0.199
Niacin (mg/d)	22.8 (10.7)	19.6 (9.9)	0.165
Vitamin A retinol eq (μg/d)	1062.9 (1116.2)	754.9 (728.9)	0.143
Vitamin C (mg/d)	213.7 (219.6)	124.8 (111.2)	0.025
Vitamin E (mg/d)	10.5 (8.1)	8.1 (4.2)	0.113
Vitamin D (μg/d)	4.4 (3.2)	4.4 (4.9)	0.966
Sodium (mg/d)	2225.06 (975.9)	2127.06 (997.5)	0.641
Potassium (mg/d)	4055.1 (2562.2)	3309.7 (2319.4)	0.159
Iron (mg/d)	15.9 (9.9)	13.4 (9.05)	0.229
Calcium (mg/d)	961.2 (471.07)	801.6 (470.6)	0.114
Phosphorus (mg/d)	1446.3 (635.4)	1274.3 (615.3)	0.201
Magnesium (mg/d)	456.1 (277.4)	381.4 (250.3)	0.191
Zinc (mg/d)	14.2 (9.4)	11.5 (6.6)	0.134
Copper (mg/d)	2.2 (1.3)	1.9 (1.3)	0.255
Selenium (μg/d)	112.5 (52.1)	106.3 (50.9)	0.573

MUFA, monounsaturated fatty acids; PUFA, polyunsaturated fatty acids; SFA, saturated fatty acids.

### 3.4 Dietary intake according to MCI status

The correlation between mean intake of major food groups and nutrients by clinical parameters are reported in Table 4. No significant associations have been identified for specific foods or nutrients with exception for alcohol intake, which inversely correlated with HDRS scores and vitamin C which directly correlated with MoCa scores.

**TABLE 4:** Spearman correlation coefficients for major food groups and nutrients, and clinical parameters in the study sample.

	MMSE	MoCa	GDS	HDRS	ESS
Fruit	0.105	0.091	0.109	−0.103	−0.061
Vegetables	0.072	0.162	−0.017	−0.051	0.032
Olive oil	−0.071	−0.076	0.131	0.124	−0.018
Legumes	0.028	0.074	−0.033	−0.029	0.082
Red meat	0.037	0.119	−0.059	0.023	0.033
Processed meat	0.173	0.063	−0.072	0.006	0.052
Fish	−0.095	−0.153	−0.126	−0.013	−0.125
Refined grains	−0.102	0.005	−0.138	−0.084	0.100
Whole grains	0.061	0.183	−0.133	−0.160	−0.008
Total grains	0.013	0.148	−0.153	−0.155	0.028
Potatoes	−0.168	−0.202	−0.141	−0.107	−0.035
Dairy	−0.150	−0.033	−0.035	0.014	−0.003
Sweets	0.058	0.076	0.095	0.077	0.041
Nuts	0.200	0.027	−0.094	−0.087	−0.052
Alcohol	0.001	−0.089	−0.115	−0.263*	−0.008
Energy (kcal)	−0.015	0.081	−0.097	−0.136	−0.065
Energy (kj)	−0.018	0.079	−0.091	−0.143	−0.057
Proteins	−0.015	0.116	−0.081	−0.121	−0.054
Lipids	0.061	0.091	−0.061	−0.043	−0.024
Cholesterol	−0.008	0.085	−0.038	−0.020	−0.024
Carbohydrates	−0.050	0.070	−0.091	−0.133	−0.083
Total fibers	0.005	0.106	−0.042	−0.151	−0.087
Total SFA (%)	0.039	0.165	0.024	−0.003	−0.004
Total MUFA (%)	0.039	0.041	−0.097	−0.058	−0.023
Total PUFA (%)	0.063	−0.021	−0.138	−0.090	−0.076
C18:3	0.139	0.067	−0.083	−0.112	−0.073
C22:6	−0.071	−0.161	−0.142	0.002	−0.121
Thiamine	0.055	0.140	0.007	−0.073	0.027
Riboflavin	0.049	0.140	0.050	−0.034	0.038
Niacin	−0.006	0.076	−0.112	−0.131	−0.046
Vitamin A (retinol eq)	0.071	0.171	0.053	−0.130	−0.087
Vitamin C	0.105	0.247*	0.035	−0.159	−0.092
Vitamin E	0.062	0.080	−0.032	−0.111	−0.064
Vitamin D	−0.074	−0.143	−0.152	0.004	−0.122
Sodium	−0.078	0.075	−0.137	−0.081	−0.087
Potassium	0.014	0.129	−0.003	−0.161	−0.085
Iron	0.019	0.118	−0.030	−0.151	−0.028
Calcium	0.017	0.193	0.036	−0.120	−0.039
Phosphorus	0.001	0.135	−0.044	−0.135	−0.037
Magnesium	0.014	0.101	−0.049	−0.156	−0.059
Zinc	0.011	0.139	−0.021	−0.149	−0.086
Copper	−0.005	0.081	−0.063	−0.153	−0.085
Selenium	−0.067	0.080	−0.166	−0.134	−0.091

MUFA, monounsaturated fatty acids; PUFA, polyunsaturated fatty acids; SFA, saturated fatty acids.

*P < 0.05.

### 3.5 Inflammatory biomarkers and cognitive status

**FIGURE 1:**
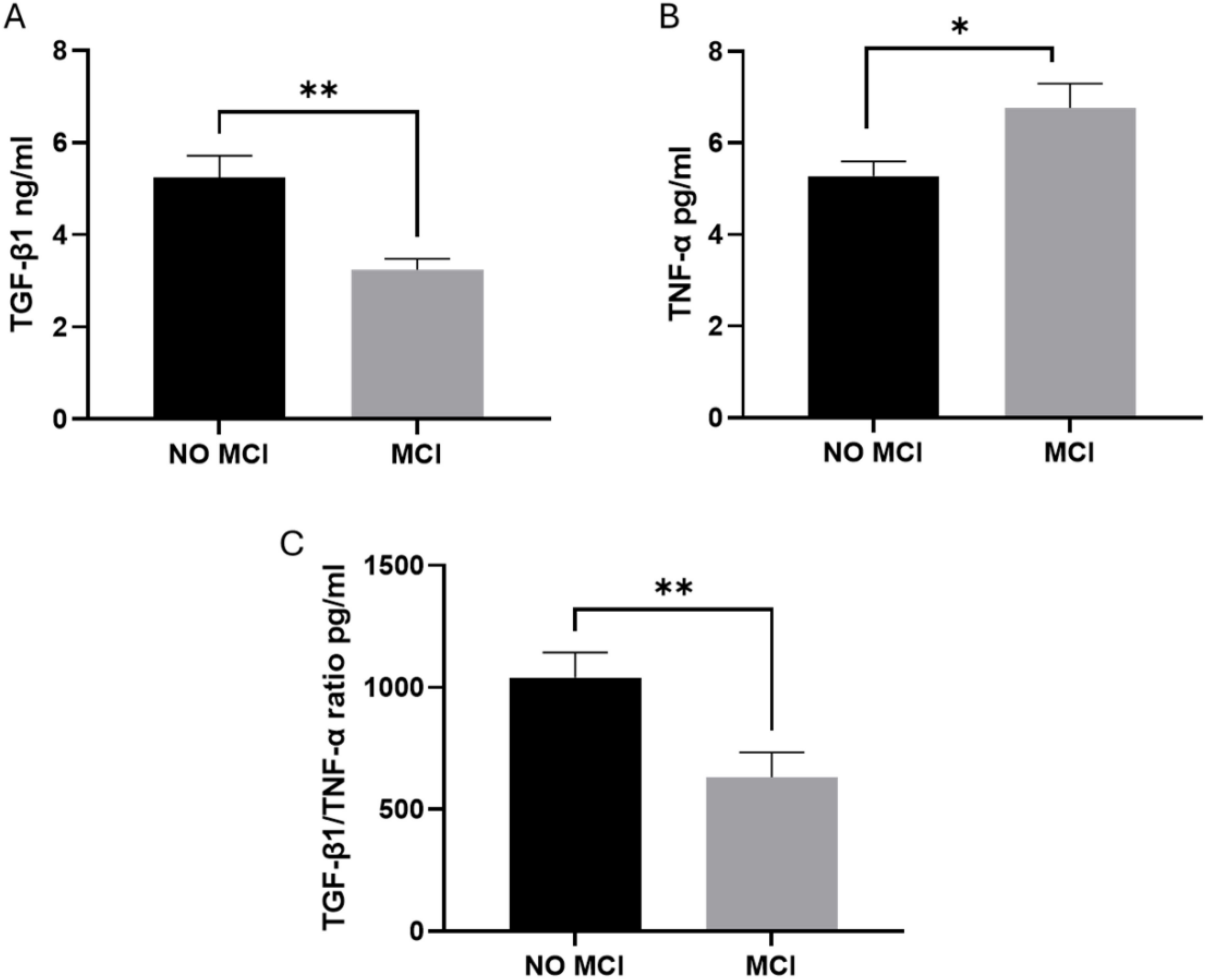
Plasmatic concentration of TGF-beta1 and TNF-alpha in MCI and non MCI subjects at the time of neuropsychological evaluation. **(A)** TGF-β1 ng/ml, **(B)** TNF-α pg/ml, and **(C)** TGF-β1/TNF-α ratio measured by ELISA assay.

As shown in Figure 1A, a deficit in TGF-β1 ng/ml plasmatic concentrations was observed in MCI subjects compared to non-MCI. A statistically significant increase of TNF-α pg/ml was also observed in MCI patients when compared to the non-MCI group (Figure 1B). As shown in Figure 1C, a marked reduction of TGF-β1/TNF-α ratio pg/ml was evident in MCI patients compared to the non-MCI group.

### 3.6 Association between Mediterranean diet adherence and likelihood of MCI

Multivariable logistic regression analyses evaluating the association between adherence to the Mediterranean diet and odds of MCI are shown in Table 5. In the model adjusted only for total energy intake (Model 1), progressively higher adherence to the Mediterranean diet was strongly associated with lower odds of MCI. Compared with the lowest adherence quartile (Q1), the odds ratios (ORs) for MCI were 0.13 (95% CI: 0.03–0.56) for Q2, 0.10 (95% CI: 0.03–0.41) for Q3, and 0.04 (95% CI: 0.01–0.21) for Q4. After additional adjustment for age, sex, smoking status, BMI, and educational level (Model 2), the inverse association remained evident, though slightly attenuated: ORs were 0.29 (95% CI: 0.05–1.74) for Q2, 0.15 (95% CI: 0.03–0.80) for Q3, and 0.07 (95% CI: 0.01–0.60) for Q4. Further adjustment for circulating inflammatory markers (TGF-β1 and TNF-α; Model 3) did not materially change the direction of the associations, with ORs of 0.29 (95% CI: 0.03–3.24), 0.13 (95% CI: 0.02–1.01), and 0.06 (95% CI: 0.00–1.10) across increasing quartiles of adherence. These findings indicate a robust dose–response relationship between higher Mediterranean diet adherence and lower likelihood of MCI, independent of major demographic and clinical confounders.

**TABLE 5:** Association between adherence to the Mediterranean diet (assessed by the Mediterranean Diet Score) and likelihood of mild cognitive impairment (MCI).

	MCI, OR (95% CI)			
Mediterranean diet	Q1	Q2	Q3	Q4
Model 1 ^a^	1	0.13 (0.03–0.56)	0.10 (0.03–0.41)	0.04 (0.01–0.21)
Model 2 ^b^	1	0.29 (0.05–1.74)	0.15 (0.03–0.80)	0.07 (0.01–0.60)
Model 3 ^c^	1	0.29 (0.03–3.24)	0.13 (0.02–1.01)	0.06 (0.00–1.10)

^a^Adjusted for energy intake.

^b^Adjusted for age, sex, smoking status, BMI, and educational level.

^c^Adjusted as model 2 + TGF-beta and TNF-alpha levels.

### 3.7 Sensitivity analyses

To evaluate the robustness of the Mediterranean diet score, sensitivity analyses were conducted by sequentially excluding individual components of the score (Supplementary Table 1). The inverse association between overall adherence and MCI persisted across most modified scores, suggesting that the observed relationship was not driven by any single food group. Additional analyses examining individual food groups in relation to MCI risk (Supplementary Table 2) did not reveal consistent or statistically significant associations after multivariable adjustment. This further supports the relevance of the overall dietary pattern rather than isolated food components in relation to cognitive status.

## 4 Discussion

In the present study, higher adherence to the Mediterranean diet was associated with a lower likelihood of mild cognitive impairment, supporting the hypothesis that overall dietary patterns, rather than isolated nutrients or foods, play a meaningful role in cognitive health during aging. Interestingly, further adjustment for inflammatory biomarkers resulted in a strengthening of the association between Mediterranean diet adherence and MCI. This pattern suggests that inflammation is associated with both diet adherence and MCI in a direction that attenuated the observed association before adjustment, resulting in a correlated, but not univocal pathway through which diet may exert potential positive effects on brain health. Importantly, the results from the sensitivity analyses reinforce the concept that the Mediterranean diet exerts its effects through the synergistic interaction of its components, rather than through the action of single dietary elements in isolation.

The findings presented in this study are consistent with a growing body of epidemiological evidence linking Mediterranean diet adherence to slower cognitive decline, and reduced risk of MCI and dementia (20). With reference to studies specifically investigating the relation between adherence to the Mediterranean diet and MCI, an early longitudinal analysis is the Washington Heights–Inwood Columbia Aging Project (WHICAP), in which Scarmeas and colleagues evaluated adherence to a 0–9-point Mediterranean diet score and subsequent cognitive outcomes in a multiethnic cohort. Among 1,393 cognitively normal participants followed for a mean of 4.5 years, those in the highest adherence tertile had a borderline lower risk of incident MCI compared with the lowest tertile (HR 0.72; 95% CI 0.52–1.00; *p* = 0.05), with evidence of a dose-response trend (p-trend = 0.05). Notably, in a separate WHICAP analysis restricted to individuals with prevalent MCI (*n* = 482), higher Mediterranean diet adherence was more robustly associated with lower risk of progression to Alzheimer’s disease over a mean of 4.3 years (highest vs. lowest tertile HR 0.52; 95% CI 0.30–0.91; *p* = 0.02), suggesting that dietary pattern adherence may be relevant both to early impairment and to downstream clinical trajectories (35). Complementary evidence comes from cross-sectional analyses in distinct settings. In the Australian Imaging, Biomarkers and Lifestyle (AIBL) study, Gardener et al. examined baseline Mediterranean diet adherence in 1,112 older adults (768 cognitively healthy controls, 133 with MCI, 211 with Alzheimer’s disease). In multivariable models, each 1-point increase in Mediterranean diet score was associated with materially lower odds of being classified as MCI (reported as ∼13–19% lower odds per unit increase, depending on model specification), supporting an inverse association between dietary pattern adherence and prevalent MCI in a geographically and culturally non-Mediterranean context. At the same time, the authors highlighted the limitations intrinsic to cross-sectional inference, including potential differential misclassification of diet among cognitively impaired participants (36). Randomized clinical studies addressing MCI as an outcome is comparatively limited but informative. Within the PREDIMED-Navarra cognitive substudy, Martinez-Lapiscina et al. assessed cognitive status after 6.5 years of dietary intervention comparing a Mediterranean diet supplemented with extra-virgin olive oil (EVOO), a Mediterranean diet supplemented with nuts, and a low-fat control diet in a subsample (*n* = 285; 95 per arm). After adjustment for demographic, vascular, and lifestyle covariates, the Mediterranean diet+EVOO group showed lower odds of MCI versus control (OR 0.34; 95% CI 0.12–0.97), whereas the Mediterranean diet+nuts group did not significantly differ from controls for MCI in the published summary. Although this substudy was not primarily powered for clinical cognitive diagnoses, it provides trial-based support that sustained enhancement of an overall Mediterranean dietary pattern (particularly in an EVOO-rich formulation) may influence clinically meaningful cognitive categorization (37). Evidence from the longitudinal investigation from the Mediterranean basin, in particular Moli-sani Study, where the Italian adults were followed for over 12 years, shows the inverse relation between higher adherence to the Mediterranean diet and low-grade inflammation (38). However, only a few other studies investigated the mediating effect of inflammation between adherence to the Mediterranean diet and cognitive outcomes. Within the WHICAP/Columbia Aging framework, Gu and colleagues explicitly tested whether reduced inflammation and improved metabolic profile could mediate the association between Mediterranean diet adherence and subsequent risk of Alzheimer’s disease (AD). In this prospective analysis (n≈1,219 non-demented adults ≥ 65 years; 4-year follow-up; 118 incident AD cases), higher Mediterranean diet adherence was associated with a more favorable biomarker profile, most consistently, lower hsCRP across adherence categories, yet the authors reported that the observed protective association of Mediterranean diet adherence with AD risk did not appear to be meaningfully mediated by hsCRP (nor by the metabolic biomarkers examined), suggesting either (i) other unmeasured inflammatory pathways (e.g., cytokine networks, cellular immune activation), (ii) cumulative/long-term inflammatory exposure not captured by a single baseline measurement, or (iii) non-inflammatory mechanisms operating in parallel (vascular, metabolic, oxidative) (39). Relatedly, in the InCHIANTI cohort (Italy), Tanaka et al. examined long-term trajectories of global cognition (MMSE) over extended follow-up and reported that higher Mediterranean diet adherence was associated with slower cognitive decline. Importantly for mechanistic interpretation, the association remained after adjustment for baseline inflammatory markers (including CRP and IL-6) and for circulating nutrient biomarkers (e.g., carotenoids, vitamin E, fatty acids), implying that the observed diet–cognition relationship was not fully explained by these measured inflammatory indices at baseline. This pattern is consistent with a scenario in which the Mediterranean diet’s neuroprotective effects reflect a broader systems-level modulation of inflammatory biology (including downstream neuroimmune effects), rather than being attributable to a single inflammatory biomarker (40). In line, recent evidence has increasingly highlighted the role of long-term adherence to other balanced dietary patterns, such as the MIND, Nordic, and DASH diets, in supporting cognitive health. Greater adherence to these dietary models has been consistently associated with improved cognitive performance (41, 42), as well as a reduced risk of dementia among older adults (43, 44). The neuroprotective effects of these dietary patterns appear to be similar to those observed for the Mediterranean diet and may be partially attributed to their high content of bioactive compounds, including polyphenols, vitamins, and unsaturated and polyunsaturated fatty acids (45). These components are known to exert antioxidant and anti-inflammatory effects, which are critical in mitigating neurodegenerative processes. In particular, the modulation of chronic low-grade inflammation, a key contributor to cognitive decline, has been proposed as a central mechanism underlying higher adherence to these dietary patterns (46, 47). Collectively, these findings support the hypothesis that adherence to nutrient-dense, anti-inflammatory dietary patterns may play a significant role in preserving cognitive function and reducing the burden of age-related neurodegenerative disorders.

Several biological mechanisms may explain the observed association between Mediterranean diet adherence and reduced likelihood of MCI, with neuroinflammatory modulation emerging as a central (but not univocal) pathway (24). Chronic low-grade inflammation is increasingly recognized as a key contributor to cognitive decline and neurodegenerative processes, including synaptic dysfunction, neuronal loss, and accumulation of pathological proteins (48). During the last years, it has been proposed a strong neurobiological link in cognitive impaired brain between an early pro-inflammatory process and a deficit of TGF-β1, a neurotrophic factor and an anti-inflammatory cytokine endowed with a neuroprotective activity against amyloid-induced neurotoxicity (49–51). In this study, we found a significant deficit in the TGF-β1 peripheral levels at the time of the neuropsychological evaluation, suggesting that this neurotrophic factor, with a key role in the memory and synaptic plasticity formation (52), could represent a new peripheral tool able to monitor the cognitive decline in its early stage, given that could reflect in the periphery the biological alterations occurring at the central level. This hypothesis could be further supported by our findings that showed a reduction of TGF-β1/TNF-α ratio in patients diagnosed by MCI compared to the other participants, and that this ratio was correlated with a reduction in MMSE and MoCa scores in MCI patients supporting the interest to evaluate its plasmatic concentration in a longitudinal evaluation in order to confirm its advantage to predict the risk of dementia-related cognitive decline in its prodromal phase. The Mediterranean diet is inherently a balanced dietary pattern, allowing adequate intakes of numerous nutrients (53) that may act additively or synergistically on shared biological pathways relevant to neurodegeneration. The Mediterranean diet is characterized by a high intake of foods with anti-inflammatory properties, such as fruits, vegetables, legumes, olive oil, and fish, which are rich in polyphenols, omega-3 fatty acids, and other bioactive compounds (54). Previous summary of evidence showed a potential decreased risk of cognitive decline associated with consumption of major polyphenol classes (55–59) and omega-3 fatty acids (60–62). These constituents have been shown to downregulate pro-inflammatory cytokines, reduce oxidative stress, and modulate immune responses at both systemic and central nervous system levels (63, 64). Importantly, while individual foods have been independently associated with cognitive benefits (65–67), focusing on single dietary components may limit the complexity of habitual dietary intake and the interactions among nutrients within a whole diet (24). The combined presence of antioxidant-rich plant foods, healthy lipid profiles driven by monounsaturated and polyunsaturated fats, and reduced intake of pro-inflammatory saturated fats may collectively amplify neuroprotective effects beyond those achievable by any single dietary component (24). Neuroinflammation is closely intertwined with oxidative stress, another critical mechanism implicated in cognitive aging (48). Reactive oxygen species can damage neuronal membranes, proteins, and DNA, thereby impairing synaptic plasticity and cognitive function (68). The Mediterranean diet provides a high antioxidant capacity through its abundance of plant-derived foods and extra-virgin olive oil, potentially enhancing endogenous antioxidant defenses and limiting oxidative damage within the brain (69). This antioxidant-inflammatory balance may be particularly relevant in the early stages of cognitive impairment, when neurobiological alterations may still be partially attenuated (70).

In addition to direct neuroinflammatory and antioxidant actions, the Mediterranean diet may indirectly protect cognitive function through its favorable impact on vascular and metabolic health (71). Cerebrovascular dysfunction, insulin resistance, and dyslipidemia are well-established risk factors for cognitive decline and MCI (72). Endothelial dysfunction is increasingly viewed as a central intermediate phenotype linking cardiometabolic risk to cerebrovascular damage and cognitive impairment, because it precedes overt atherosclerosis and is associated with impaired vasodilatory capacity, increased vascular inflammation, blood–brain barrier vulnerability, and reduced cerebral perfusion (73). Higher adherence to the Mediterranean diet has been consistently associated with improved endothelial function, reduced atherosclerotic burden, better glycemic control, and healthier lipid profiles (74). These vascular benefits translated to cognitive function are likely driven by the combined effects of multiple characteristic components of the diet, including extra-virgin olive oil, omega-3 polyunsaturated fatty acids (PUFAs) from fish, dietary fiber, and a wide range of plant-derived (poly) phenols (75, 76). Extra-virgin olive oil, particularly rich in monounsaturated fatty acids and phenolic compounds such as hydroxytyrosol and oleuropein, has been demonstrated to improve endothelial-dependent vasodilation, reduce oxidative modification of LDL particles, and decrease circulating markers of vascular inflammation (77, 78). Similarly, omega-3 PUFAs derived from regular fish consumption [particularly eicosapentaenoic acid (EPA) and docosahexaenoic acid (DHA)] have been shown to exert potent vascular and neurovascular benefits with improved endothelial function (79). DHA is a structural component of neuronal membranes and cerebral vasculature, while both EPA and DHA promote nitric oxide–mediated endothelial function, reduce platelet aggregation, and attenuate vascular inflammation (80). Polyphenols from fruits, vegetables, legumes, nuts, and red wine constitute another key pathway linking the Mediterranean diet to brain vascular health: compounds such as flavonoids, resveratrol, and anthocyanins enhance endothelial nitric oxide bioavailability, reduce oxidative stress within the vascular wall, and inhibit inflammatory signaling cascades (81, 82). Summary evidence of the literature has shown that higher polyphenol intake improves flow-mediated dilation and microvascular function as well as control blood pressure (83, 84). Given the strong association between endothelial dysfunction and white matter damage, cerebral small-vessel disease, and cognitive impairment, these polyphenol-mediated effects represent a plausible biological bridge between Mediterranean dietary habits and preserved cognitive status. Given the strong links between vascular pathology and cognitive impairment, these cardiometabolic benefits likely contribute substantially to the observed association with lower MCI prevalence.

An additional mechanism that may contribute to the association between Mediterranean diet adherence and lower likelihood of MCI involves modulation of the gut microbiota (85) and the gut-brain axis (86). The Mediterranean diet is rich in dietary fibers, polyphenols, and unsaturated fatty acids, all of which are key determinants of gut microbial composition and function (87). High intake of fruits, vegetables, legumes, whole grains, and nuts provides fermentable substrates that promote the growth of beneficial microbial taxa, including short-chain fatty acid (SCFA)–producing bacteria such as *Faecalibacterium prausnitzii* and *Roseburia* species (87). SCFAs, particularly butyrate, exert anti-inflammatory effects, maintain intestinal barrier integrity, and have been shown to influence microglial activation, neuroinflammation, and synaptic plasticity (88, 89). Furthermore, higher intakes of fermented foods with probiotic effects may positively influence gut homeostasis (90). Polyphenols abundant in extra-virgin olive oil, fruits, vegetables, nuts but also in culinary herbs (91) further interact bidirectionally with the gut microbiota (92). While polyphenols shape microbial diversity by acting as prebiotics and selectively inhibiting pathogenic bacteria and stimulating beneficial species (93), microbial metabolism of polyphenols generates bioactive metabolites with enhanced bioavailability and neuroprotective properties (94). These metabolites may cross the blood-brain barrier and modulate signaling pathways involved in oxidative stress, inflammation, and neuronal survival (95). Moreover, circulating metabolites may also affect vascular function and improve endothelial health (96, 97). In contrast, dietary patterns high in saturated fats and refined sugars (components largely minimized in the Mediterranean diet) have been associated with gut dysbiosis, increased intestinal permeability, and systemic inflammation (98), all of which may exacerbate neuroinflammatory processes implicated in cognitive decline (99). Through these microbiota-mediated pathways, the Mediterranean diet may influence cognitive function both indirectly, by reducing peripheral inflammation and metabolic dysfunction, and directly, via neuroimmune and neurochemical signaling along the gut–brain axis (100). Some evidence from observational and clinical studies support such a hypothesis, although additional studies are needed to further confirm the contribution of the gut microbiota on cognitive brain health (101). This emerging evidence supports the notion that the neuroprotective effects of the Mediterranean diet extend beyond classical antioxidant and vascular mechanisms, reinforcing the importance of dietary pattern–driven regulation of gut microbiota in the prevention or delay of early cognitive impairment (101).

Some limitations should be considered when interpreting these findings, including the observational nature of the study, which precludes causal inference, and the potential for residual confounding by lifestyle or socioeconomic factors associated with healthier dietary behaviors. Additionally, dietary adherence was self-reported, which may introduce measurement error. Nonetheless, the observed association is biologically plausible and consistent with prior literature, lending robustness to the findings.

In conclusion, the present results support the hypothesis that higher adherence to the Mediterranean diet is associated with a lower likelihood of mild cognitive impairment. Overall, the present findings align with the existing literature in suggesting that higher adherence to a Mediterranean dietary pattern is associated with a lower likelihood of MCI and, in some cohorts, a lower risk of progression along the cognitive impairment continuum. However, the variability in effect estimates across studies likely reflects differences in dietary scoring methods, baseline cardiometabolic risk profiles, cultural dietary contexts (including the absolute consumption of hallmark foods such as olive oil), and the clinical rigor and consistency of MCI adjudication. The findings underscore the importance of considering the synergistic effects of dietary patterns and highlight neuroinflammatory protection, along with vascular and metabolic pathways, as key mechanisms linking diet to cognitive health. These considerations strengthen the rationale for interpreting the association in terms of a dietary pattern effect (capturing synergistic and cumulative exposures) while also motivating harmonized prospective studies and adequately powered randomized trials with standardized MCI endpoints. Future longitudinal and interventional studies are warranted to further clarify causality, identify critical windows of exposure, and determine whether improving adherence to the Mediterranean diet can effectively prevent or delay the onset of cognitive impairment in aging populations.

## Data Availability

The data that support the findings of this study are available upon reasonable request.
