# Supplementary material for: Adherence to the Mediterranean diet, inflammatory biomarkers and cognitive status in older Italian adults
Source: Front Nutr. 2026 Apr 29;13:1809163. doi: 10.3389/fnut.2026.1809163 (PMC13170460; doi:10.3389/fnut.2026.1809163)
Supplement: Supplementary file 1 [file Table_1.docx]

***Supplementary Material***

**Supplementary Figure 1.** Study design scheme.


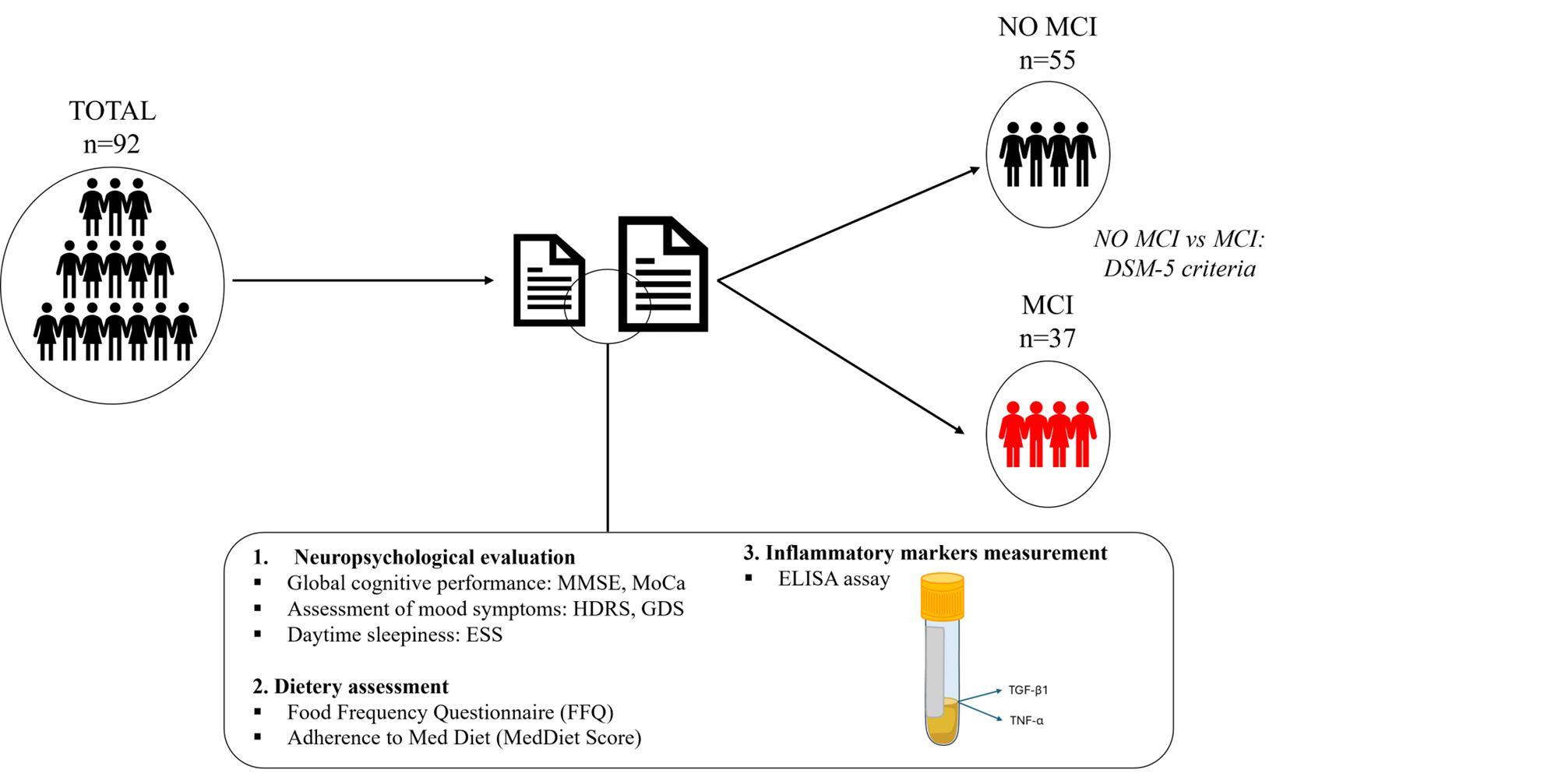


MCI, Mild Cognitive Impairment; MMSE, Mini Mental Examination; MoCA, Montreal Cognitive Assessment; HDRS, Hamilton Depression Rating Scale; GDS, Geriatric Depression Scale; ESS, Epworth Sleepiness Scale; FFQ, Food Frequency Questionnaire; ELISA, Enzyme-Linked Immunosorbent Assay; TGF-β1, Transforming Growth Factor-beta1; TNF-α, Tumor Necrosis Factor-alpha.

**Supplementary Table 1.** Sensitivity analyses of the association between Mediterranean Diet Score and likelihood of mild cognitive impairment after sequential exclusion of individual score components.

|  | MCI, OR (95% CI)^a^ | | | |
| --- | --- | --- | --- | --- |
|  | Q1 | Q2 | Q3 | Q4 |
| MDS - cereals | 1 | 0.21 (0.02-2.11) | 0.26 (0.03-1.99) | 0.05 (0.00-0.72) |
| MDS - potatoes | 1 | 0.45 (0.04-4.68) | 0.20 (0.03-1.46) | 0.06 (0.00-0.77) |
| MDS - fruit | 1 | 0.04 (0.00-0.54) | 0.21 (0.02-2.00) | 0.03 (0.00-0.55) |
| MDS - vegetables | 1 | 0.21 (0.01-3.25) | 0.19 (0.03-1.33) | 0.12 (0.01-1.12) |
| MDS - legumes | 1 | 0.21 (0.03-1.52) | 0.20 (0.03-1.56) | 0.06 (0.00-0.80) |
| MDS - fish | 1 | 0.16 (0.01-2.00) | 0.14 (0.01-1.26) | 0.08 (0.01-0.72) |
| MDS - red meat products | 1 | 0.32 (0.04-2.90) | 0.05 (0.00-0.62) | 0.05 (0.00-0.85) |
| MDS - poultry | 1 | 0.30 (0.03-3.22) | 0.13 (0.02-0.97) | 0.07 (0.00-0.96) |
| MDS - dairy products | 1 | 0.09 (0.01-0.87) | 0.21 (0.02-2.50) | 0.05 (0.00-0.48) |
| MDS - olive oil | 1 | 0.11 (0.01-1.51) | 0.20 (0.03-1.35) | 0.06 (0.01-0.90) |
| MDS - alcohol | 1 | 0.30 (0.04-2.40) | 0.09 (0.01-1.15) | 0.08 (0.01-0.69) |
| ^a^ adjusted for energy intake, age, sex, smoking status and educational level, TGF-beta and TNF-alpha levels | | | | |

**Supplementary Table 2.** Association between consumption of major food groups characteristic of the Mediterranean diet and likelihood of mild cognitive impairment (MCI).

|  | MCI, OR (95% CI)^a^ | | | |
| --- | --- | --- | --- | --- |
|  | Q1 | Q2 | Q3 | Q4 |
| Potatoes | 1 | 0.63 (0.04-10.76) | 0.89 (0.04-18.81) | 0.20 (0.01-4.68) |
| Fruit | 1 | 1.25 (0.13-12.36) | 0.32 (0.02-4.08) | 1.58 (0.16-15.52) |
| Vegetables | 1 | 1.18 (0.12-11.59) | 0.38 (0.04-4.08) | 0.95 (0.09-9.90) |
| Legumes | 1 | 4.04 (0.52-31.53) | 0.23 (0.01-4.06) | 0.76 (0.11-5.44) |
| Fish | 1 | 2.26 (0.21-24.70) | 0.12 (0.01-1.88) | 6.09 (0.41-90.52) |
| Eggs | 1 | 3.60 (0.36-35.74) | 2.35 (0.28-19.74) |  |
| Refined grains | 1 | 0.93 (0.11-7.98) | 3.55 (0.26-47.68) | 0.92 (0.11-7.63) |
| Whole grains | 1 | 0.36 (0.05-2.38) |  |  |
| Total grains | 1 | 2.11 (0.24-18.47) | 0.22 (0.02-2.49) | 0.36 (0.03-4.33) |
| Red meat products | 1 | 0.15 (0.01-3.15) | 0.14 (0.02-1.20) | 0.61 (0.07-5.45) |
| Processed meat | 1 | 1.18 (0.11-13.00) | 2.70 (0.36-20.17) |  |
| Dairy products | 1 | 1.16 (0.13-10.34) | 1.37 (0.16-11.85) | 0.43 (0.05-3.58) |
| Sweets products | 1 | 0.52 (0.06-4.21) | 0.66 (0.07-6.66) | 1.46 (0.14-15.14) |
| Nuts | 1 | 1.12 (0.09-13.65 | 1.59 (0.13-19.48) | 2.57 (0.24-27.53) |
| Olive oil | 1 | 0.16 (0.02-1.56) |  |  |
| Alcohol | 1 | 1.28 (0.18-9.03) | 0.68 (0.07-6.16) |  |
| ^a^ adjusted for energy intake, age, sex, smoking status and educational level, TGF-beta and TNF-alpha levels | | | | |
